# Cross-cultural adaptation, content validity, and reliability of the Amharic version of the modified John-Hopkins fall risk assessment scale among older adults who attend home health care services

**DOI:** 10.3389/fpubh.2024.1470517

**Published:** 2024-11-27

**Authors:** Samuel Teferi Chanie, Moges Gashaw, Kassaw Belay Shiferaw, Fiseha Sefiwu Zinabu, Setegn Fentahun, Kassahun Cherkos, Alemu Kassaw Kibret, Ermias Solomon Yalew, Assefa Kebad Mengesha, Habtamu Semagne Ayele, Zufan Yiheyis Abriham, Mihret Dejen Takele

**Affiliations:** ^1^Department of Physiotherapy, College of Medicine and Health Science, University of Gondar, Gondar, Ethiopia; ^2^Department of Psychiatry, College of Medicine and Health Science, University of Gondar, Gondar, Ethiopia; ^3^Department of Pharmacology, School of Pharmacy, College of Medicine and Health Science, University of Gondar, Gondar, Ethiopia; ^4^Department of Medical Parasitology, School of Biomedical and Health Sciences, College of Medicine and Health Science, University of Gondar, Gondar, Ethiopia

**Keywords:** Amharic, content validity, cross-cultural adaptation, home health care service, modified John-Hopkins fall risk assessment scale, older adults

## Abstract

**Background:**

The modified John-Hopkins fall risk assessment tool (mJH-FRAT) is a comprehensive and multi-factor fall risk assessment tool used to screen and grade older adult’s fall risk levels in home health care services. This can help to identify risky individuals early, establish prevention protocols, and reduce the occurrence of injury. Nevertheless, there is a dearth of contextually valid and reliable fall risk assessment tools among this population in the study area. The aim of this study is therefore to cross-culturally adapt and assess the content validity and reliability of the modified John-Hopkins fall risk assessment tool among older adults following home health care in Ethiopia.

**Method:**

The English version of the mJH-FRAT underwent cross-cultural adaptation into Amharic. The final Amharic version was subjected to face validity and then content validity was computed. This community-based study was conducted from November 2023 to May 2024 with a total of 150 participants selected through convenience sampling. Data collection occurred through face-to-face interviews. Epi-Info 7 and Statistical Package for the Social Sciences software version 25 facilitated data entry and analysis, respectively. Reliability was assessed by employing intra-rater and inter-rater reliability using Cohen’s kappa.

**Result:**

The CVI based on the item level of all the items was between 0.8 and 1. The S-CVI based on average for domains such as general condition and clinical condition was 0.925 and 1, respectively, and the S-CVI (average) of the scale was 0.96. The S-CVI based on the universal agreement value for the overall 8 items was 0.75. The kappa statistic coefficient value was between 0.79 and 1. The intra-rater reliability and inter-rater reliability were 0.94 and 0.93, respectively.

**Conclusion:**

The rigorous adaptation process, face and content validity, and reliability analyses demonstrated that the Amharic mJH-FRAT is a content valid and reliable tool for assessing the fall risk level in this population. Clinicians and researchers can utilize this tool for the advancement of both clinical practice and research work on this group of people in Ethiopia.

## Introduction

### Background and statement of the problem

Injuries are a significant public health concern worldwide (1, 2) and it became the fourth leading cause of death among older adults (3). According to data from Ethiopia’s Health and Demographic Surveillance Sites, injuries constitute 6.4% of the 9,719 older adult deaths recorded in 3 years, with fall down injuries accounting for the majority (4). Now a days, fall down injuries have become one of the most prevalent and harmful injuries that affect older adults (5).

Age-related physical changes, pre-disposing comorbidities, and environmental factors like utilizing worn out shoes are usual causes of fall among the older adults (6). Regardless, a fall down injury might be fatal or non-fatal. Non-fatal fall injuries are highly correlated with a loss of independence (7) and it contributes to increased health care expenditures and a lower quality of life among older adults, and the expenses along with the health care services related to falls at older ages are rising dramatically on a global base (8).

Fall down injuries account for multiple complications, including fractures, among the older adult population (9). Comparably, it takes much longer for older adults to recover from fall-related injuries owing to diminishing functional capacity and physiological deterioration associated with age than adults (10, 11). Falls can lead to long-term disabilities and physical dependence (12). Falls cause not only physical injuries but also psychological repercussions such as a sense of unhappiness and discomfort (13).

Since falling exacerbates the chance of a recurrence of falling and is related to further detrimental health effects, including fear of falling (14, 15). Fear of falling, the level of certainty that someone feels in their ability to execute activities of daily living (ADL) without falling, by itself hinders the ability to perform routine activities for living, increasing the degree of dependence on assistance and reducing one’s autonomous (16). Moreover, falling increases the likelihood of falling after a certain period of time and corresponds to deconditioning, fragility, and gait disturbance (17). Commonly, surgical management, physiotherapy, and psychotherapy interventions are included in managing fall complications (18).

There are a number of fall risk assessment tools to measure the incidence of falls among community-dwelling older adults; however, fall risk level assessment among older adults following home health care services is untouched in the country. The John-Hopkins fall risk assessment tool (JH-FRAT) was first developed by Stephanie S. et al. to examine multi-factor fall risks among older adults in acute health care settings (19).

To apply JH-FRAT in the community for older adults following home health care services, the modified John-Hopkins fall risk assessment tool (mJH-FRAT) was cross-culturally adapted and validated (20) and it has demonstrated high specificity and sensitivity and is easy to use (20). The tool has seven items that help predict an older adult’s fall risk level. It has a total score of 35 and three categories, such as low risk (0–6), moderate risk (7–13), and high risk (14–34).

Taking into consideration the incidence of falls among older adults following home health care services and its negative impact on their ADL, psychological status, and quality of life, assessing their fall risk level with a valid and reliable measurement tool is a crucial step. Moreover, the measurement tool’s psychometric properties should be evaluated contextually to avoid its varying nature in relation to cultural context, literacy level, and age. However, there is no cross-culturally adapted, valid, and reliable fall risk measurement tool in Amharic to use among such a population in Ethiopia. Therefore, the aim of the present study is to cross-culturally adapt and examine the content validity and reliability of the English version of the modified John-Hopkins fall risk assessment tool on this population in Ethiopia.

## Method

### Study procedure and period

This community-based cross-cultural adaptation, content validation and reliability study was conducted from November, 2023 to May, 2024. The current study followed a two-stage methodology to accomplish its objective. First, translation and cross-cultural adaptation of the English mJH-FRAT into Amharic, face and content validity were carried out. Next, the reliability of the Amharic version of mJH-FRAT was scrutinized. This study was carried out based on the Helsinki Declaration. The Institutional Review Board of the School of Medicine of the University of Gondar approved the study (ref no: SOM 575). The signed informed consent form was attained from every participant after providing a verbal and written account. Stage one:

### Translation and cross-cultural adaptation into Amharic

The translation and cross-cultural adaptation of the English mJH-FRAT into Amharic language occurred based on the cross-cultural adaptation and translation of measurement tool guideline stated by Beaton’s (21). The detailed procedural steps are mentioned below.

Step 1: Two bilingual English-speaking forward translators who are fluent in both Amharic and English independently translated the original mJH-FRAT along with its specific instructions into Amharic (Am). As recommended, the first forward translator had a medical background, and he was from the department of physiotherapy at the school of medicine and health sciences at the University of Gondar, and he had information about the aim of the study. Whereas the second forward translator was an English language expert, and he is from the English language department, and he had no information about the aim of the study.

Step 2: The two forward translators and principal investigator combined the two forward translations (FT1 and FT2) into a common version (FT12) through consensus. This step resulted in a consensus translation of the Amharic version of mJH-FRAT (mJH-FRAT-Am I), and the principal investigator was there to mediate the synthesis of T12.

Step 3: Forward-translated Amharic version I of mJH-FRAT was translated back into English by two bilingual back translators independently. The two translators were neither aware nor informed about the concepts explored to avoid information bias and highlight unexpected meaning (22). Both the translators were from the department of English language, at the University of Gondar. They met and synthesized the back-translated English version of mJH-FRAT. This process was to make sure the content of the back-translated version was consistent with the content of the original version and spotting out possible imperfections.

Step 4: The expert panel was formed by five members: three physiotherapists (MSc), one from the language department (MSc), and one methodologist (PHD). The panel members, therefore, met and reviewed the translated version and discussed the clarity, understandability, and comprehensiveness of all the items in the questionnaire to reach a consensus on any possible discrepancy. Moreover, the panelists assessed the equivalence of the original and translated versions using four criteria: semantic equivalence, idiomatic equivalence, experiential equivalence, and conceptual equivalence (23). Finally, they proposed what would be considered the pre-final version of the questionnaire for further testing.

Step 5: The pre-testing of the pre-final Amharic version of mJH-FRAT was carried out using cognitive interviews with participants from the target population. The cognitive interview was carried out with a total of 30 older adults who follow home health care services in the study area. These participants were involved for the pre-testing purpose only. Hence, they did not participate in the reliability assessment. The clarity of instructions, language appropriateness, cultural suitability, and acceptability of the scale were evaluated as well. Lastly, the translated and cross-culturally adapted Amharic version of mJH-FRAT was prepared for face and content validation assessment.

### Face validity

Face validation is performed to assure that lay experts easily understand the items of the scale in terms of their feasibility, formatting, readability, and/or clarity in language (24). The adapted modified John-Hopkins fall risk assessment tool was checked for face validity by 15 randomly selected lay experts with yes or no responses to indicate favorable and unfavorable evaluation criteria, respectively. Lay experts were health practitioners (physiotherapists and clinical nurses) who were providing health care services at home. They were asked if the format and items were clear, understandable, and contextual language selection.

### Content validity

Content validity is also known as intrinsic validity, and it is a pre-request for further statistical validity. It is used to measure the comprehensiveness and representativeness of the content domain of items in a questionnaire (25). Moreover, it allows the researchers to obtain a clear picture of the limitations, dimensions, and components of the construct from the panel of experts (26). The content validity of the adapted mJH-FRAT was assessed by content validity determination and judgment quantification (27).

Two independent expert panels were formed for the content validity determination and quantification procedure. Each expert panel had 10 members from academia (physiotherapy, occupational therapy, and biostatistics departments). The criteria for panel member selection were expert’s knowledge of the subject matter, specific training, and work experience over 5 years. A consent form was sent to all the experts, and they were notified that they were taking part in the study voluntarily and that they had the right to withdraw at any time. The adapted modified John-Hopkins fall risk assessment questionnaire was then sent to the members of the expert panels through email after their consent to participate was secured.

The first expert panel was responsible for content validity determination. Additionally, they were also responsible for adding, removing, or modifying the items and evaluating the scale’s items for their representativeness, applicability, and feasibility in low-resource study settings. A scheduled face-to-face panel discussion was held to reach a consensus regarding their judgments to review and endorse the appropriate and feasible means of instruction.

The second expert panel was formed mainly to reduce over-or under-estimation of rating and judging. The panel members received the tool with a checklist to rate the preliminary scale’s items in terms of their relevancy and essentiality for the content domain of the scale. The rating process took no more than 15 min.

#### Content validity determination

The content validity determination was conducted using both developmental and judgmental stage (28). The development stage comprises three steps, such as domain identification, item generation, and instrument construction (29, 30). The domain identification was done using a literature review, content analysis, and panel expert suggestions (31). One item was added during the item generation step. The items were then arranged in each domain, reworded as suggested, and refined by the panel experts for the final scale construction.

#### Content validity quantification

Content validity index based on item level (I-CVI), scale level content validity index based on average agreement (S-CVI/Ave), scale level content validity index based on universal agreement (S-CVI/UA), content validity ratio, and Kappa statistic coefficient (K) were employed for the quantification of the content validity of the adapted modified John-Hopkins fall risk assessment tool.

#### Content validity index (CVI)

It is the procedure that enables raters to independently review and score the relevance of the items to the content domain represented by the tool (27). The CVI for each individual item (I-CVI) as well as for the total scale (S-CVI) was computed. The relevance category has four points, such as: not relevant = 1, somewhat relevant = 2, quite relevant = 3, and highly relevant = 4. The CVI is the proportion of a score of 3 or 4 given to the items by the experts (27).

The content validity index value for individual items ranges from 0 to 1, and I-CVI > 0.79 is considered the item being relevant for the content domain of the scale (32, 33). The scale level content validity index has two approaches, such as based on the average (S-CVI/Ave) and based on universal agreement (S-CVI/UA). The acceptable value of scale level CVI based on average (S-CVI/Ave) and universal agreement (S-CVI/UA) values was set at 0.8 and 0.7, respectively (34).

#### Kappa statistic coefficient

The kappa statistic shows the percentage of agreement that remains after a chance agreement is taken out. The total amount is compared with the highest value that may be achieved, which allows for agreements that arise only by chance, considering the distribution of the marginal item ratings allocated by each expert (35, 36). The kappa statistic, a consensus index of inter-rater agreement, is added to CVI to ensure that the chance agreement has no effect on the expert agreement (31). Kappa’s assessment criteria is that values above 0.74 are considered excellent (37).

#### Content validity ratio (CVR)

In accordance with Lawshe’s principle, the content validity ratio is the ratio of the number of experts rating the items of the tool as essential to the total number of expert panel members. It assesses if the items of the tool are necessary to conduct a certain construct by observing a set of experts who rated each of the items in terms of a three-point scale, such as 1 = essential, 2 = useful but not essential, and 3 = not essential (38).

When an item is rated as “essential” by all experts, the CVR value will be equal to 1, the CVR value will be between 0 and 1 when the number of respondents who rate the item as “essential” is greater than half yet less than all, and the value of the CVR will be negative if less than 50 % of the experts rate the item as “essential” (39). The acceptable value of CVR was set at 0.6 and above (39).

### Phase two: reliability assessment

#### Study setting

The present study was scrutinized to translate and cross-culturally adapt the English modified John Hopkins fall risk assessment tool into Amharic and assess the face validity, content validity, and inter-rater and intra-rater reliability of the Amharic version among older adults following home-based health care in Ethiopia. Although Ethiopia has multiple ethnic groups that speak different languages (40), Amharic is its official and national language, and it is also the first language in the study area, Gondar city. Gondar is the ancient and largely populated city, and it is located nearly 800 km north of Addis Ababa, the capital of Ethiopia. The city has six sub-cities with 25 Kebeles.

#### Study population, inclusion and exclusion criteria

This study included older adult individuals who follow home based health care service in Gondar city during the study period. The inclusion criteria were older adults who attend health care interventions at their home for at least two visits per week, who had a willingness to participate in the study, who can walk with or without assistive devices, and who are able to speak and understand Amharic. This study excluded bed-ridden older adults, who are medically diagnosed with psychological disorders or cognitive impairment, since they may not respond appropriately. The eligibility criteria screening began after the individual’s willingness to participate in the study was secured.

#### Sample size and sampling technique

There is still a dearth of precise and universal sample size determination technique for the validity and reliability assessment (41). The sample size determination for the reliability assessment studies is usually between 20 individuals for the 3-point rating scale and 100 for the 7-point scale. The recommended minimal sample size for the 4, 5, and 6-point scales is 30, 50, and 75, respectively (42). This study assumed 150 older adults for the reliability assessment (75 for inter-rater and 75 for intra-rater reliability). The convenience sampling method was employed to select the study participants.

#### Data collection

Well-trained physical therapy professionals (MSc) were engaged in the data collection process. The signed informed consent form was obtained from every participant after providing a verbal and written account before proceeding with data collection. Additionally, the participants were given a concise explanation regarding the purpose of the study and that their personal information was going to be kept confidential.

One trained professional collected data twice from the same respondent with 2 weeks of duration in between one session and the other to avoid recall bias for the intra-rater reliability evaluation. On the other hand, two trained professionals collected data from the same respondent one after the other within 15 min of rest in between for the inter-rater reliability assessment. Data collectors who participated during the inter-rater reliability assessment had no access to the other collector’s results to prevent bias. The principal investigator and other co-authors strictly reviewed the data for clarity, accuracy, and completeness.

#### Reliability analysis

Reliability evaluates how well a certain measurement tool has a consistent result. Additionally, reliability assessment aids in identifying mistakes in content sampling, variances in respondents’ characteristics, and preferences for measurement scales (43). The reliability of the Amharic version of mJH-FRAT was assessed by intra-rater and inter-rater reliability.

Intra-rater reliability examines the consistency of rating scores given by the same rater over time (44). On the other hand, inter-rater reliability is the degree to which various raters provide consistent estimates of the same construct. It assesses an agreement among two or more raters (45).

The intra-rater and inter-rater reliability assessments of Am-JHFRAT were computed using Cohen’s kappa coefficient (K) since it is categorical. The value of kappa ranges from 0 to 1. A Cohen’s kappa value of 0 indicates no agreement between the two rates. 0.01–0.20 indicates poor agreement, 0.21–0.40 indicates slight agreement, 0.41–0.60 indicates fair agreement, 0.61–0.80 indicates good agreement, 0.81–0.92 indicates very good agreement, 0.93–1.0 indicates excellent agreement, and 1.0 indicates perfect agreement between two rates (45).

Epi-Info 7 data program and Statistical Package Social Science (SPSS) version 25 software were used to enter and analyze the data, respectively. The participant’s socio-demographic characteristics were reported by descriptive statistical analysis using counts (*n*) and percentages (%) through texts and tables.

## Results

### Translation and cross-cultural adaptation into Amharic

A robust procedure was followed to translate and cross-culturally adapt the original mJH-FRAT into Amharic. The forward and backward translations were carried out satisfactorily. Next, an in-person discussion was held by the expert committee to screen the establishment of equivalence between the translated and the original questionnaire based on the criteria mentioned earlier. Then, the committee approved that the translated questionnaire fulfilled all the equivalence criteria. Moreover, the instructional design was endorsed to be a face-to-face questionnaire administration method to make the tool feasible for the illiterate people. In conclusion, the translation and cross-cultural adaptation task was completed successfully.

### Face validity

The present study involves 15 lay experts for face validity assessment, and they reported that all the items were easy to understand. Additionally, they also asserted that the domain frame of the questionnaire was logical. Moreover, the experts support that the scale format was relevant to the measuring tool. Conversely, they revealed a jargon phrase that would be difficult to understand by the study population. The phrase in the item (ሲራምዱ ሚዛንዎን ለመጠበቅ ይቸገራሉ) was replaced with more clear words (ሲራምዱ የመንገዳገድ ችግር አለብዎት ወይ) as lay experts have recommended. Generally, the Amharic mJH-FRAT demonstrates excellent face validity.

### Content validity

The content validity assessment included a total of 20 panel experts in two different phases. All the experts approved the two identified domains and all the items in the first phase of the panel discussion. The panelists raised invaluable comments and added one item (“presence of fear of falling”) that is evidenced to be related to the content domain of the questionnaire and a cause for falling (46). Moreover, there was some modification in terms of item relocation to another domain and reordering of items with their respective domains. Then, the revised and adjusted version was resubmitted via email to experts for approval. Finally, the preliminary Amharic version of the modified John-Hopkins fall risk assessment tool, composed of eight items with two domains, was prepared for content validity quantification.

The content validity quantification phase shows that the item level CVI of all the items in both domains ranged from 0.8 to 1 (Table 1). The S-CVI based on average for the overall items was 0.96. The S-CVI based on the universal agreement value for the overall 8 items was 0.75. The kappa statistic coefficient value was ranged from 0.79 to 1 (Table 1). The CVR result was between 0.8 and 1 (Table 1).

**Table 1:** Content validity quantification of the Amharic version of the modified John-Hopkins fall risk assessment tool among older adults following home-based health care service in Ethiopia, 2024.

Domain	Items	I-CVI	UA	Pc	K	CVR
General condition	Age	1	1	0.000976	1	1
	History of fall in the past 6 months	1	1	0.000976	1	1
	Fear of fall	1	1	0.000976	1	1
	Physical activity status	1	1	0.000976	1	1
Comorbidities	Medication	1	1	0.000976	1	1
	External appliance	0.9	0	0.00976	0.899	1
	Incontinence	0.8	0	0.044	0.79	0.8
	Alertness	1	1	0.000976	1	1

CVR, content validity ratio; I-CVI, item level content validity index; K, kappa statistic coefficient; Pc, probability of chance agreement and UA, universal agreement.

The above findings support that all the items are relevant and essential for the content domain of the questionnaire, and the expert’s agreement was not affected by a chance agreement. This implies that the Am-JHFRAT is highly content valid to measure fall risk levels among older adults receiving home based health care services in Ethiopia.

### Socio-demographic data for reliability analysis

The current study included a total of 150 older adult participants (75 for intra-rater reliability and 75 for inter-rater reliability) for the reliability assessment. The majority (43.4%) of the participant’s ages were between 70 and 75 years old. Above half (67.4%) of them were male. Only a few (12%) of participants achieved a college or higher education level. Additionally, the majority (62.7%) of them were married. Over half (68%) of them were Orthodox Christians. Furthermore, the majority (46%) of them had a moderate level of fall risk. The detailed socio-demographic data is illustrated in the Table 2.

**Table 2:** Socio-demographic data of the participants for the reliability assessment of the Amharic version of John-Hopkins fall risk assessment tool among older adults following home-based health care service in Ethiopia, 2024.

Socio-demographic data	Category	Frequency (*n*)	Percentage (%)
Gender	Female	49	32.6
	Male	101	67.4
Age	65–70	62	41.4
	70–75	65	43.4
	>75	23	15.2
Educational status	Unable to read and write	64	42.7
	Primary school	37	24.7
	Secondary school	31	20.7
	College and above	18	12
Marital status	Married	94	62.7
	Divorced	29	19.3
	Widowed	27	18
Religion	Orthodox Christian	102	68
	Muslim	28	18.7
	Catholic	11	7.3
	Protestant	9	6

%, percentile; *n*, frequency.

### Reliability

The overall intra-rater reliability of the Amharic version of the modified John-Hopkins fall risk assessment scale was excellent (*K* = 0.94). In addition, the Cohen’s kappa coefficient value for the intra-rater reliability of each domain was 0.90 and 0.86, respectively. The result reveals that the overall tool has excellent intra-rater reliability and at the domain level as well.

Similarly, the overall inter-rater reliability of the Amharic version of the modified John-Hopkins fall risk assessment scale was excellent (*K* = 0.93). Moreover, the Cohen’s kappa coefficient values for the inter-rater reliability of each domain were 0.89 and 0.92, respectively, which supports the fact that the tool has excellent inter-rater reliability both at the scale and domain level.

## Discussion

This study was carried out to translate and cross-culturally adapt the modified John-Hopkins fall risk assessment tool into Amharic and to evaluate the content validity and reliability of the Amharic modified John-Hopkins fall risk assessment tool in an Ethiopian context for use during home-based health care services. Fall risk level assessment among this population has been neglected so far, and no related study has been done before in the country. Hence, the current study addressed this particular gap for a comprehensive fall risk assessment and selective management.

During the cross-cultural adaptation process, additional words were included for further clarification of each item to make the instruction clear. Generally, the translators reached consensus later with a principal investigator as a mediator, and the overall adapting process was done satisfactorily. During the face validation, few rewordings were made on the specific item that resembled jargon words to enhance the item’s understandability for the participants by adhering to the lay expert’s suggestions. In conclusion, the questionnaire demonstrated satisfactory face validity.

The content validity assessment procedure was carried out in two independent phases to avoid expert’s over-and under-estimation bias. Experts added one item (“the presence of fear of falling”) that can be a cause for the incidence of falls among older adults (46, 47) during the first phase of the content validity procedure. Additionally, they also proposed and approved two domains, namely, general conditions and clinical conditions. Moreover, they reordered and arranged the items in their respective domains. Furthermore, the type of data collection method was suggested to be structured face-to-face questionnaire administration by considering the educational background of the majority of the study population.

Evidence asserts that there cannot be a single set of guidelines for establishing a defensible cut point for the scores of all tests (48). Thus, the one item added was made to hold its own score out of 2. Therefore, the total score was made out of 30, and the grade classification was established as follows: low fall risk (< 11 total points), moderate fall risk (11–22 total points), and high fall risk (>22 total points). Moreover, the content validity quantification finding supports that the Am-JHFRAT is highly content-valid in the Ethiopian context.

The values of the intra-rater and inter-rater reliability were 0.94 and 0.93, respectively, which together suggests that the Am-JHFRAT has excellent intra-rater and inter-rate reliability to assess the fall risk level of older adults who follow home-based clinical services in Ethiopia. This content valid and reliable scale is easy to use, and the items are well related to the content domain of the scale. Thus, it can be applied during home health care services for the early detection of older adult’s fall risk levels and to proceed and apply targeted interventions to prevent post-complications secondary to fall injuries (19).

### Strength and limitation of the study

The present study provides a contextual, content valid, and reliable fall risk assessment scale to apply during home-based health care services. The face validity was also evaluated, followed by a content validity examination. The study finding supports its use in the advancement of home based health care services and research work on this population to classify their fall risk level.

Despite this, the current study has a few limitations. To begin with, the study utilized a relatively small sample size with a convenient sampling technique, which may affect the representativeness of the result. Furthermore, due to the fact that there is no similar validated tool that measures the construction under consideration in an Ethiopian context, we were not able to scrutinize the tool’s construct and criterion validity, suggesting a need for further psychometric assessment of this measurement tool in future studies for better evidence with a large sample size and probability random sampling technique.

## Conclusion and clinical implication

The robust translation, cross-cultural adaptation process, face and content validity examination, and reliability examination support that the Amharic version of JH-FRAT is a content valid and reliable scale to assess the fall risk level of older adults following home-based health care services. This valid and reliable Am-JHFRAT is a time saver and easy to use for the advancement of home health care services and research works. This in turn helps health care providers detecting older adult’s fall risk levels early, take specific and targeted interventions, and reduce the incidence of fall-down injury post-complications (49) (Supplementary File).

Generally, understanding older adult’s fall risk level through a valid and reliable assessment tool and applying comprehensive prevention measurement promotes the older adult’s quality of life and happy aging and reduces the individuals and public’s economic burden. Further studies including disaggregated analysis are welcomed to solidify the finding on male and female participants.

## Data Availability

The original contributions presented in the study are included in the article/supplementary material, further inquiries can be directed to the corresponding author.

## Supplementary material

Supplementary File 1 The cross-culturally adapted and validated Amharic version of the modified John-Hopkins fall risk assessment questionnaire.
