# Supplementary material for: Cross-cultural adaptation, content validity, and reliability of the Amharic version of the modified John-Hopkins fall risk assessment scale among older adults who attend home health care services
Source: Front Public Health. 2024 Nov 27;12:1470517. doi: 10.3389/fpubh.2024.1470517 (PMC11631703; doi:10.3389/fpubh.2024.1470517)
Supplement: Supplementary File 1 — The cross-culturally adapted and validated Amharic version of the modified John-Hopkins fall risk assessment questionnaire. [file Supplementary_file_1.docx]

**የተሻሻለው ጆን ሆፕኪንስ የመውደቅ አደጋ መገምገሚያ መጠይቅ አማረኛ እትም**

የተሻሻለው ጆን ሆፕኪንስ የመውደቅ አደጋ መገምገሚያ መጠይቅ ቤታቸው ውስጥ ሆነው የህክምና ክትትል የሚያደርጉ አረጋውያን ምን ያክል ለመውደቅ አደጋ ተጋላጭ እንደሆኑ ለማዎቅ ያገለግላል፡፡ በአጠቃላይ መጠይቁ ሁለት ክፍሎች አሉት፡፡ አንደኛው የአረጋውያኑን አጠቃላይ ሁኔታ የሚያስስ ሲሆን ሁለተኛው ደግሞ የአረጋውያኑን የተጓዳኝ የጤና እክል ሁንታ ይዳስሳል፡፡

**የመውደቅ አደጋ መገምገሚያ ነጥብ ስሌት፡**

በእያንዳንዱ ጥያቄ ውስጥ ተገቢውን ምርጫ ያስመርጡ፡፡ እንደሁም ከአንድ በላይ ምላሽ ላላቸው ጥያቄዎች ነጥባቸውን በመደመር ያስቀምጡ፡፡ የጥያቄው ምላሽ ^“^የለም^”^ ከሆነ የጥያቄው ነጥብ 0 ይሆናል፡፡

| ተ.ቁ | ጥያቄ | | ነጥብ |
| --- | --- | --- | --- |
|  | አጠቃላይ ሁኔታ | |  |
| 1 | እድሜዎ ስንት ነው? | 1) 60-69 አመት(1 ነጥብ)  2) 70-79 አመት (2 ነጥብ)  3) 80 አመት እና ከዚያ በላይ (3 ነጥብ) |  |
| 2 | በ6 ወራት ውስጥ ቢያንስ አንድ ጊዜ ወድቀው ያውቃሉ ወይ? | 1) አዎ (5 ነጥብ)  2) የለም (0 ነጥብ) |  |
| 3 | የመውደቅ ፍራቻ አለብዎት ወይ? | 1) አዎ (2 ነጥብ)  2) የለም (0 ነጥብ) |  |
| 4 | የአካላዊ እንቅስቃሴዎት ሁኔታ እንዴት ነው  (ከ አንድ በላይ ምርጫ መምረጥ ይቻላል)? | 1) ለመንቀሳቀስ ወይም ከቦታ ቦታ ለመዛወር እርዳታ ወይም ክትትል ያስፈልግዎታል? (2 ነጥብ)  2) ሲራምዱ እግርዎ ይንገዳገዳል? (2 ነጥብ)  3) የመንቀሳቀስ ችሎታዎን የሚጎዳ የእይታ ወይም የመስማት ችግር አለብዎት? (2 ነጥብ)  4) የለም (0 ነጥብ) |  |
|  | **የጤና እክሎች** | |  |
| 5 | ለመውደቅ አደጋ ተጋላጭ የሚያደርጉ መድሃኒት ይዎስዳሉ ወይ? | 1) አዎ አንድ መድሃኒት እወስዳለው (3 ነጥብ)  2) አዎ ሁለት መድሃኒት እወስዳለው (5 ነጥብ)  3) የእንቅልፍ መድሃኒት እወስዳለው (7 ነጥብ)  4) የለም (0 ነጥብ) |  |
| 6 | ስንት የታካሚ መንከባከቢያ መሳሪያዎችን ይጠቀማሉ? | 1) አንድ (1 ነጥብ)  2) ሁለት (2 ነጥብ)  3) ሶሰት እና ከዛ በላይ (3 ነጥብ)  4) የለም (0 ነጥብ) |  |
| 7 | ሽንት እና ሰገራ ሲፀዳዱ ምን ያስቸግርዎታል? | 1) ሽንት እና ሰገራ መቆጣጠር አለመቻል (2 ነጥብ)  2) አጣዳፊ ወይም ተደጋጋሚ ሽንት እና ሰገራ (3 ነጥብ)  3) አጣዳፊ የሆነ ሽንት እና ሰገራ እና መቆጣጠር አለመቻል (4 ነጥብ)  4) የለም (0 ነጥብ) |  |
| 8 | የንቃትዎ ሁኔታ መን ያክል ነው?  (ከ አንድ በላይ ምርጫ መምረጥ ይቻላል) | 1) ፈጣን የአካባቢ ለውጥ ግንዛቤ እጥረት አለብዎት ወይ? (1 ነጥብ)  2) አልፎ አልፎ አንዳንድ አላስፈላጊ ተግባሮችን ያለምክኒያት ያከናውናሉ ወይ? (2 ነጥብ)  3) የእርስዎን አካላዊ እና አዕምሯዊ እክል ግንዛቤ እጥረት አለብዎት ወይ? (4 ነጥብ)  4) የለም (0 ነጥብ) |  |

በመጠይቁ መሰረት ጠቅላላ ድምር ውጤቱ ከ 10 በታች ከሆነ ዝቅተኛ የመውደቅ አደጋ ፤ በ 11 እና 22 መካከል ከሆነ ደግሞ መካከለኛ የመውደቅ አደጋ እና ከ 22 በላይ ከሆነ ከፍተኛ የመውደቅ አደጋ እንዳለው ያመላክታል፡፡ እባክዎ ሁሉንም ነጥቦች በመደመር ቤታቸው ውስጥ ሆነው የህክምና ክትትል ለሚያደርጉ አረጋውያን የመውደቅ አደጋ ደረጃቸውን ለይተው ያስቀምጡ፡፡

የጥናቱ ተሳታፊ ስለሆኑ ከልብ እናመሰግናለን!
